# Dominant and novel clades of *Candidatus* Accumulibacter phosphatis in 18 globally distributed full-scale wastewater treatment plants

**DOI:** 10.1038/srep11857

**Published:** 2015-07-03

**Authors:** Yanping Mao, David W. Graham, Hideyuki Tamaki, Tong Zhang

**Affiliations:** 1 Environmental Biotechnology Laboratory, Department of Civil Engineering, The University of Hong Kong, Pokfulam Road, Hong Kong; 2 School of Civil Engineering and Geosciences, Newcastle University, Newcastle upon Tyne NE1 7RU, United Kingdom; 3 Bioproduction Research Institute, National Institute of Advanced Industrial Science and Technology (AIST), Tsukuba, Japan

## Abstract

Here we employed quantitative real-time PCR (qPCR) assays for polyphosphate kinase 1 (*ppk1*) and 16S rRNA genes to assess relative abundances of dominant clades of *Candidatus* Accumulibacter phosphatis (referred to Accumulibacter) in 18 globally distributed full-scale wastewater treatment plants (WWTPs) from six countries. Accumulibacter were not only detected in the 6 WWTPs performing biological phosphorus removal, but also inhabited in the other 11 WWTPs employing conventional activated sludge (AS) with abundances ranging from 0.02% to 7.0%. Among the AS samples, clades IIC and IID were found to be dominant among the five Accumulibacter clades. The relative abundance of each clade in the Accumulibacter lineage significantly correlated (p < 0.05) with the influent total phosphorus and chemical oxygen demand instead of geographical factors (e.g. latitude), which showed that the local wastewater characteristics and WWTPs configurations could be more significant to determine the proliferation of Accumulibacter clades in full-scale WWTPs rather than the geographical location. Moreover, two novel Accumulibacter clades (IIH and II-I) which had not been previously detected were discovered in two enhanced biological phosphorus removal (EBPR) WWTPs. The results deepened our understanding of the Accumulibacter diversity in environmental samples.

Enhanced biological phosphorus removal (EBPR) processes promote the removal of phosphate from wastewater, which can reduce eutrophication in receiving water bodies. Polyphosphate-accumulating organisms (PAOs), which are promoted in EBPR processes, take up extraneous phosphate and store it as intracellular polyphosphate under cyclic anaerobic and aerobic conditions. In most lab-scale reactors, especially those fed with short-chain fatty acids like acetate, propionate and pyruvate as the primary carbon sources, a guild of microbes named *Candidatus* Accumulibacter phosphatis (henceforth referred to Accumulibacter) have been identified as key to the EPBR process^1^. Accumulibacter are affiliated with the *Rhodocyclus* group in β-*Proteobacteria* and also are widely found in full-scale wastewater treatment plants (WWTPs)^2^,^3^,^4^. However, Accumulibacter have proven difficult to isolate in pure culture even when such strains have been enriched to over 90% abundance as indicated by fluorescence *in situ* hybridization (FISH)^5^.

Given that Accumulibacer is not readily culturable, genetic approaches are needed for assessing its prevalence in treatment processes. However, clusters in Accumulibacter cannot be clearly defined by 16S rRNA genes since they shared high identities of 16S rRNA genes (over 97%)^6^. Therefore, it was concluded to use ecotypes that contain strains with similar ecological niches for identification^7^,^8^, although they differed in certain abilities to accumulate phosphorus and reduce nitrate^4^,^9^. Specifically, the single-copy gene encoding polyphosphate kinase 1 (*ppk1*), which catalyzes the reversible reaction of polyphosphate formation from ATP, was chosen as a high resolution biomarker to differentiate relative divergence among Accumulibacter ecotypes. Based on the phylogenetic distance of the *ppk1* genes, the Accumulibacter lineage had been subdivided into five clades in Type I and seven clades in Type II^8^,^10^,^11^. Within this context, quantitative real-time PCR (qPCR) assays for detecting the Accumulibacter 16S rRNA and *ppk1* genes were developed by He *et al.*^8^ according to the DNA sequences extracted from environmental samples. These assays allow one to quantify and characterize Accumulibacter lineages in samples, which overcome low detection limits of quantitative FISH caused by strong auto-fluorescence and weak signals due to the low activity of PAOs, and had been used to examine abundances of Accumulibacter in WWTPs^8^,^12^,^13^ and dominant Accumulibacter clades in lab-scale bioreactors^14^.

Clearly, approaches for studying Accumulibacter are improving and the local-scale knowledge is growing about these organisms in WWTPs. For example, Peterson *et al.*^11^ characterized the Accumulibacter lineage structure in WWTPs performing EBPR in two regions of USA (Wisconsin and California) and found that WWTP habitat characteristics, but not geographic distance, appeared to be determining dominant Accumulibacter lineage. Alternately, Albertsen *et al.*^12^ characterized the microdiversity of Accumulibacter in a Danish EBPR WWTP according to detected *ppk1* sequences and FISH results. However, both these studies only assessed Accumulibacter lineages at EBPR WWTPs from narrow geographic regions; i.e., they did not assess Accumulibacter in WWTPs on a global scale, which is essential for understanding general relationships between Accumulibacter lineage structure and operating conditions, and to uncover the microbial diversity of Accumulibacter ecotypes.

We hypothesize Accumulibacter inhabit in activated sludge (AS) systems of diverse configurations besides EBPR WWTPs and some Accumulibacter clades remain undiscovered. Therefore, we took AS samples from 18 globally distributed full-scale WWTPs in Asia (China, Hong Kong, Singapore, and Japan), North America (Canada and USA) and Europe (UK). Of the 18 plants, 6 were indicated as EBPR and 11 were non-EBPR AS systems based on their configurations (Table 1). To address the questions of “what are the dominant and novel Accumulibacter clades in full-scale AS?” and “which operational factors affect the distribution of Accumulibacter clades?” We used qPCR to quantify Accumulibacter 16S rRNA and *ppk1* genes to investigate the relative abundances of Accumulibacter clades (Table S1).

## Results

### qPCR efficiency and accuracy

The AS samples for DNA extraction were collected from 18 WWTPs, each WWTP was assigned a code consisting of the acronyms of country-city-name of the WWTP (Table 1). To determine whether the extracted genomic DNA contained contaminants that inhibit qPCR, the sludge samples UK-WL-OW, JP-STD-TK, CN-WH-LW, JP-A2O-TK and UK-NW-NW were diluted in series and amplified by using primer sets targeting Accumulibacter clades IA, IIA, IIB, IIC and IID respectively to calculate the PCR efficiency (Figure S1). The results showed that the standard curves had correlation coefficient of 0.98–0.99 and PCR efficiencies of 93%–106%, demonstrating that the qPCR assay was accurate and PCR inhibitors in the extracted DNA were negligible.

To confirm the specificity of qPCR assays targeting Accumulibacter *ppk1* genes in specific clades, two representative qPCR products amplified by each *ppk1* gene primer set were cloned and sequenced. The phylogenetic trees recruiting qPCR product sequences and their closest GenBank matches demonstrated the specificity of the qPCR-*ppk1* assays applied in this study (Figure S2). Nucleotide sequences from qPCR products were clustered with its appropriate reference *ppk1* genes most of which had been defined by He *et al.*^8^.

### Detection of Accumulibacter abundance

The fractional abundances of the total Accumulibacter lineage relative to the bacterial community have been estimated by three approaches, i.e., 1) *ppk1* genes and bacterial 16S rRNA genes; 2) Accumulibacter 16S rRNA genes and bacterial 16S rRNA genes; and 3) pyrosequencing reads assigned to Accumulibacter and bacteria. The first two approaches were determined by qPCR, whereas the third approach was based on the 16S rRNA gene pyrosequencing (Table 2). Both the finished genome of Accumulibacter Clade IIA UW-1 (referred to CAP IIA UW-1)^15^ and the recently reported draft genomes of Clades IA^14^,^16^, IB^17^, IC^16^, IIC^16^ and IIF^16^ carried the single copy of *ppk1* gene. CAP IIA UW-1 carried two copies of *rrn* operon. Therefore, we estimated the fractional abundance of Accumulibacter by assuming that Accumulibacter genome had single copy of *ppk1* gene and 2 copies of the *rrn* operon while the bacterial genomes carried 4 copies of *rrn* operon according to the available 2,671 finished genomes in IMG/M database at the time of writing.

Total Accumulibacter abundances determined by qPCR using the *ppk1* primer set displayed similar trends to qPCR-based estimates using the Accumulibacter 16S primer set (paired Student t test, *P* = 0.08) and, to a lesser extent, 16S rRNA gene pyrosequencing data (paired Student t test, *P* = 0.38). However, estimates based on the latter two approaches were significantly different (paired Student t test, *P* = 0.03). The differences between 16S rRNA gene qPCR-based and pyrosequencing-based estimation were due to the different biases existing within each of the two quantification approaches.

According to the qPCR-*ppk1* assays, AS samples from SG-SG-UP, CN-WH-LW, UK-WL-OW, UK-NW-NW, UK-LB-LB, CN-GZ-DT and JP-A2O-TK had the highest abundances of Accumulibacter lineage among the 18 AS samples, ranging from 1.2% to 7.0%. Five of these top seven plants were indicated to promote biological phosphorus removal with anaerobic/anoxic/aerobic (A/A/O) or anoxic/aerobic and return activated sludge (A/O + RAS) fermentation configurations. The abundance of the Accumulibacter lineage identified in the AS from JP-A2O-TK was about two times higher than that from JP-STD-TK, which treated the same wastewater using different treatment processes. This can be reasonably explained by the fact that A/A/O processes (JP-A2O-TK) often to enrich Accumulibacter relative to conventional AS processes.

### Distribution of Accumulibacter clades

The relative abundance of the five major Accumulibacter clades in each WWTP was assessed by qPCR using the specific *ppk1* primer sets designed by He *et al.*^8^. For this assessment, we adopted the primer set targeting the clade IIC *ppk1* genes, excluding the NS D3 clone (accession number EF559355), because the NS D3 clone sequence was only distantly affiliated to other sequences in clade IIC and it had overlap regions with those in other clades. This may significantly affect the quantification of clade IIC by qPCR^8^,^13^.

Accumulibacter clades IIC & IID dominated the five Accumulibacter clades in 11 out of the 18 AS samples with relative abundances ranging between 30% and 99% of the total Accumulibacter lineage (Table 2) and 0.01%–1.45% of the total bacterial community (Table S2). It should be born in mind that the Accumulibacter genome of clade IID has not been obtained, although the genome retrieving work is ongoing. Besides the five clades, some other clades co-existed in the Accumulibacter lineage, which had been demonstrated by Peterson *et al.*^11^. Given that most of the Accumulibacter clades shared highly similar 16S rRNA genes, we assumed abundances of the Accumulibacter lineage in bacterial communities quantified by the qPCR-16S assay had contained the Accumulibacter of all clades. Besides those five clades whose *ppk1* genes can be amplified by the qPCR-*ppk1* assay, large proportions of other clades were estimated in the Accumulibacter lineage of over 50% abundances in 10 WWTPs, especially over 80% in sludge samples of CN-HK-ST, CN-QD-TD and CN-HK-SH (Table S3). Designing qPCR primer sets targeting more clades, for example, clades IB, IC, ID and IE in type I and clades IIE and IIF in type II^11^ based on their divergent regions in *ppk1* sequences, is necessary to reveal the distribution of different Accumulibacter clades more accurately.

**Figure 1:**
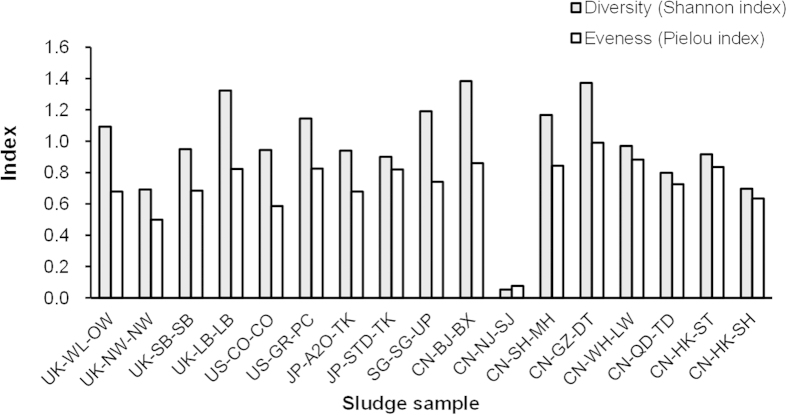
Diversity and evenness of the Accumulibacter lineage in the full-scale WWTPs indicated by Shannon index and Pielou index respectively. The indices were calculated from the relative abundances of the five Accumulibacter clades (normalized to total Accumulibacter *ppk1* genes).

To compare the Accumulibacter clade diversity and evenness among the full-scale AS samples, the Shannon diversity index and Pielou evenness index were calculated using the relative abundances of the five Accumulibacter clades as determined by qPCR targeting the *ppk1* genes (Table 2 and Fig. 1). The AS from CA-GP-GP was not included in this analysis because no Accumulibacter lineage was detected by qPCR. The least diversity and evenness were found in CN-NJ-SJ with 99% of Accumulibacter in clade IID. Notably, CN-BJ-BX and SG-SG-UP, UK-LB-LB and UK-WL-OW showed highest level of diversity, i.e., all five clades were detected and clades were more evenly distributed than in the other samples. The high diversity and evenly distribution of the Accumulibacter lineage present in these four samples might be due to the complex configuration (e.g. A/A/O + MBR in CN-BJ-BS, CAS + MBR in SG-SG-UP, A/O + RAS in UK-LB-LB, A/A/O in UK-WL-OW) and EBPR practice in the WWTPs. Moreover, among the four WWTPs in United Kingdom, only Loughborough WWTP and Wanlip WWTP were EBPR plants while the other two were conventional AS plants at the time of sampling, thus to explain why UK-LB-LB and UK-WL-OW have Accumulibacter clades in both higher abundance and diversity.

### Effects of operational variables on composition of the Accumulibacter lineage

**Figure 2:**
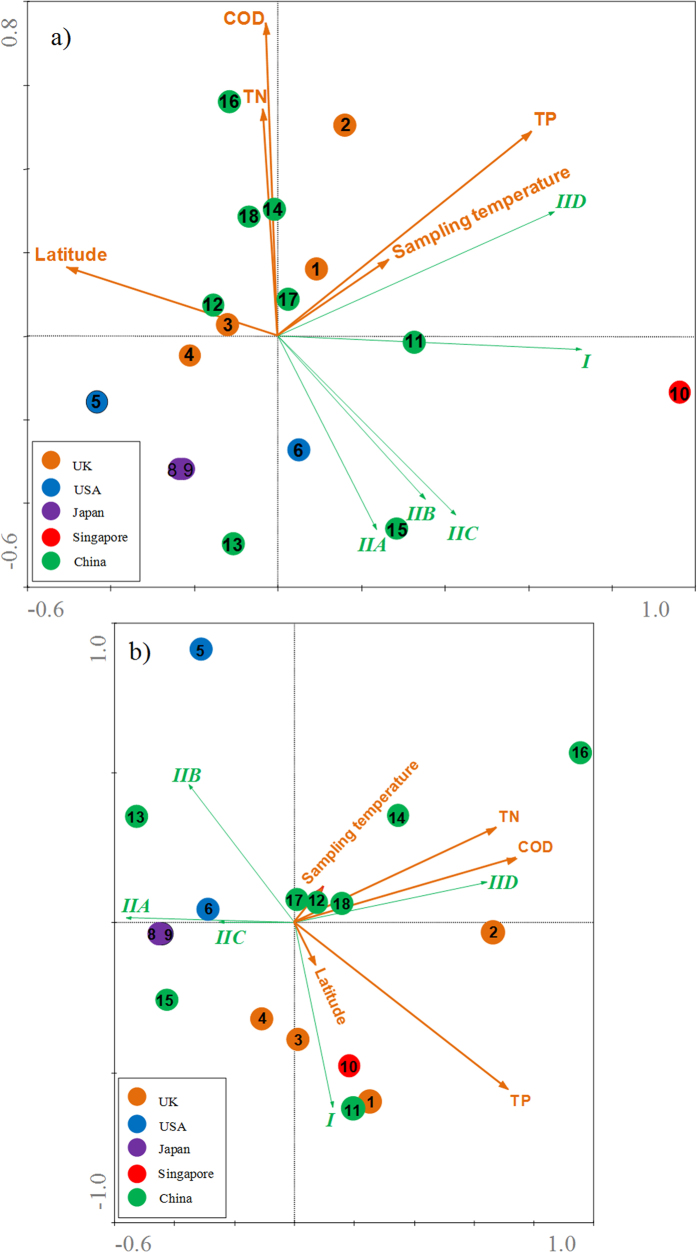
Correlation triplots based on redundancy analysis (RDA) displayed the variations of relative abundances of Accumulibacter specific-clade in **a**) the bacterial communities and **b**) the Accumulibacter lineage, basing on the qPCR assays targeting *ppk1* genes and bacterial 16S rRNA genes, with respect to the variable operational conditions among wastewater treatment plants. Each Accumulibacter clade is represented in green line, each activated sludge sample is indicated by a color point. Operational variables are indicated by orange lines.

According to the old microbiological tenet ‘everything is everywhere, but the environment selects’^18^, the lineage structure may be affected by the stimuli from WWTP operation. To further understand the effects of WWTP operating conditions on compositions of the Accumulibacter lineage, redundancy analysis (RDA) was conducted by recruiting the relative abundances of the Accumulibacter clades in the bacterial community (Fig. 2a) and within the Accumulibacter lineage (Fig. 2b). Given those clades kept quite large distance from the variable lines in Fig. 2a, the total Accumulibacter levels in bacterial communities did not significantly correlate with any single operating variable (P > 0.05) (Table S4). However, the relative abundances of specific clades in the Accumulibacter lineage showed significant correlation (P < 0.05) with WWTP influent levels of total phosphorus (TP) and chemical oxygen demand (COD) (Fig. 2b and Table S5). The proportions of the dominant clade IID in Accumulibacter were most positively influenced by influent TP, COD and total nitrogen (TN). The results were consistent with well-recognized conclusions that the level of PAOs was related to the operational variables of influent biodegradable COD, reactor configuration and amount of oxidized nitrogen entering the anaerobic zone^2^. Compared to the wastewater characteristics the geographic factors such as latitude played little role in distributing the Accumulibacter clades.

### Novel clades of the Accumulibacter lineage

**Figure 3:**
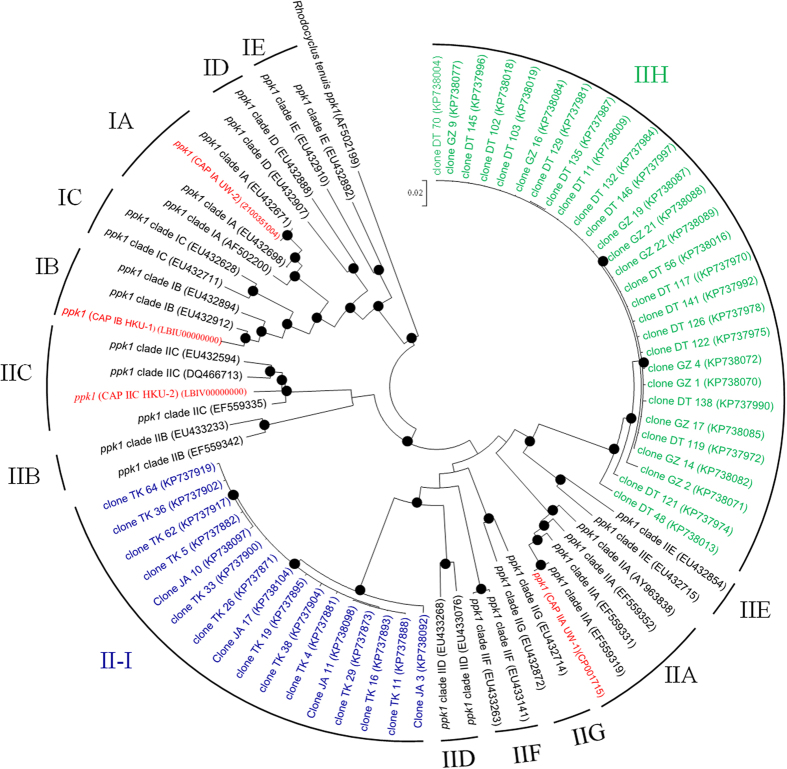
Phylogenetic tree indicating inferred relatedness of *ppk1* genes from Accumulibacter by employing maximum likelihood method. The sequences produced by *ppk1* gene clone libraries of CN-GZ-DT sludge sample were colored by green, and those of JP-A2O-TK sludge sample were colored by blue. The sequences in red are derived from draft genome sequences rather than clones. Black circles on node branches indicate >60% bootstrap support. The *ppk1* gene of *Rhodocyclus tenuis* was adopted as the most closely related outgroup sequence.

The Accumulibacter *ppk1* genes were sequenced from PCR amplicons obtained from four selected AS samples carrying out EBPR. All the retrieved sequences from UK-LB-LB and UK-WL-OW were affiliated with previously recognized Accumulibacter clades^8^,^11^, but 28 *ppk1* sequences from CN-GZ-DT and 16 *ppk1* sequences from JP-A2O-TK were relatively distant from known clades (Fig. 3 and Figure S3). These *ppk1* sequences of two novel clades (IIH and II-I) shared identities of 88% with their closest *ppk1* gene matches in Type II respectively. The observation of the novel Accumulibacter clades in EBPR AS systems expand our understanding of the diversity of its ecotypes and implied that different wastewater characteristics or operational parameters may induce the proliferation of different Accumulibacter clades.

## Discussion

We used qPCR assays to quantify the abundances of the Accumulibacter lineage in AS samples of 18 full-scale WWTPs. 6 of the 18 plants were indicated as having conditions to promote EBPR. Overall, Accumulibacter were determined to habitat in 17 AS samples with percentage of 0.02%–7.0% in bacterial communities.

Based on currently available qPCR primer sets that target *ppk1* genes, the clades IIC and IID dominated the Accumulibacter lineage of 11 of the 18 AS samples. Moreover, deeper analysis suggests that clade IIC abundances are probably underestimated by our qPCR-*ppk1* assay due to the exclusion of the NS D3 clone. Among the detected Accumulibacter clades, WWTP influent TP, COD and TN levels were positively correlated with the relative abundance of clade IID, whereas all of them were inversely correlated with clade IIC, implying functional divergence between the two clades. Some operational variables regarding to be meaningful for EBPR, e.g. solid retention time and effluent TP, were not included in the analyses, which may affect the correlation of Accumulibacter distribution and WWTPs operation to a certain extent. However, this is still a valuable starting point for understanding dominant Accumulibacter clades in full-scale WWTPs.

Consistent with previous results^11^,^12^, higher Accumulibacter diversities were apparent among the AS samples (compared with the five clades). Thus we suspect the five qPCR primer sets understand the actual abundances of Accumulibacter clades within the WWTPs. Moreover, two novel Accumulibacter clades (IIH and II-I) were identified from two full-scale AS samples, respectively. Therefore, designing new qPCR primer sets targeting more clades of *ppk1* genes, according to their divergent regions in sequences, is urgently needed.

Overall, this study for the first time shows Accumulibacter abundances and dominant clades on a global scale among various types of WWTPs. The results deepen our understanding in the distribution and diversity of the uncultured Accumulibacter guild in a large scale and provide a baseline for new studies on PAOs in other environmental scenarios.

## Methods

### Samples from WWTPs

AS samples were collected from 18 full-scale WWTPs located in seven regions (mainland China, Hong Kong, Singapore, Japan, USA, Canada, and UK) on three continents. Some important characteristics of these WWTPs are summarized in Table 1. Out of the 18 AS samples, 12 samples were previously analyzed using 454-pyrosequencing to reveal their bacterial diversity^19^. In the current study, we performed qPCR analysis to more specifically investigate the distribution of specific clades of Accumulibacter.

### DNA extraction

DNA extraction was performed in duplicate from each sludge sample, following methods reported previously^19^, using FastDNA SPIN Kit for Soil (MP Biomedicals, Solon, OH, USA). DNA purity was checked by spectrometry at 260 nm and 280 nm measured by NanoDrop^®^ Spectrophotometer ND-100 (Thermo Fisher Scientific, USA), and the DNA integrity was visualized by gel electrophoresis using λ DNA-HindIII Digest (Takara, Japan) and DL2000 (Takara, Japan) DNA markers. For accurate quantification by qPCR, DNA samples in high purity were extracted, which could be indicated by the 260/280 ratios of 1.84 ± 0.07.

### qPCR conditions

The qPCR assays quantified the abundances of Accumulibacter *ppk1* genes and 16S rRNA genes from Accumulibacter and total bacteria using primer sets published previously^8^. Thermal cycling and fluorescence detection of the qPCR were conducted on a BioRad iCycler (v. 5.0, BioRad, Hercules, CA) in a 25 μL reaction volume containing 5 ng DNA template and using SYBR Premix Dimer Eraser^TM^ (Takara, Japan). The thermal cycling protocol was as follows: initial denaturation at 95 °C for 30 s, followed by 40 cycles of denaturation at 94 °C for 5 s, annealing for 45 s, and extension at 72 °C for 30 s. The cycle threshold (Ct) was determined by setting the threshold at a relative fluorescence unit value of 5.0. The annealing temperature and specific primer concentration had been optimized to improve the quality of qPCR (Table S1).

Five- to seven-point calibration curves for the qPCR were generated by 10-fold serial dilution of plasmid carrying *ppk1* gene fragments of specific clades. Plasmids as the standard for qPCR were generated from appropriate clones. In detail, the PCR product of *ppk1*- or 16S rRNA-target genes was purified using quick-spin PCR Product Purification Kit (iNtRON, Korea) and cloned using pMD18-T Vector (TaKaRa, Japan). The plasmid then was extracted from the clone and its mass concentration was determined by NanoDrop^®^ Spectrophotometer ND-100 (Thermo Fisher Scientific, USA). Copy number was calculated based on the molecular weight and the mass concentration of each plasmid^20^,^21^.

### Quality control of qPCR

A negative control containing sterile water was used for each assay to check possible contamination and primer-dimer formation. All amplification products were visualized by agarose gel electrophoresis, some gel images could be found in Figure S4. PCR amplification efficiency (E) was estimated from the slope of the standard curve by the formula E = 10 ^-1/slope^ -1. To check the qPCR assay variation, standard curves were run in duplicate reactions within each plate. Samples were run on at least two plates with triplicate reactions per plate. Standard deviations were calculated from the average for the runs. In the present study, only those qPCR results with standard curves of R^2^ within 0.97–1.00, Ct values from 12 to 32, and PCR efficiencies of 87%–106% were accepted as the reliable data. In order to evaluate the reliability of qPCR identification and whether DNA extract of sludge samples contained PCR inhibitors, PCR efficiency values of several DNA extracts from AS samples were also checked^22^,^23^. To validate the specificity of the qPCR assays, representative qPCR products of each Accumulibacter clade were cloned and sequenced. The phylogenetic distances of the sequences and their closest GenBank matches were visualized by using MEGA (v. 5.02)^24^.

### Quantification of total Accumulibacter lineage

The abundance of the Accumulibacter lineages was examined by two qPCR assays targeting the 16S rRNA gene and the *ppk*1 gene, respectively. From this data, the fraction of Accumulibacter was estimated by assuming that one Accumulibacter genome carries two copies of the 16S rRNA gene and one copy of the *ppk*1 gene according to the finished genome CAP IIA UW-1^15^. It was assumed that other bacteria contained an average of 4.0 copies of the 16S rRNA gene per genome based on the 2,671 finished genomes in IMG/M database (downloaded on April 7, 2014)^25^.

Previous 16S rRNA gene pyrosequencing targeted the V4 regions on samples from 12 WWTPs. Given the limited number of current databases, the 16S rRNA gene of the candidate genus Accumulibacter only can be detected by the BLASTN Best-hit method based on similarity searches (not by other methods like RDP^26^ and MEGAN-LCA annotation^27^,^28^). Therefore, following the methodology published before^29^,^30^, to quantify the total Accumulibacter 16S rRNA genes, representative 16S rRNA gene sequences of various Accumulibacter clades ( ≥1200 bp) were chosen to form a specialized sub-database (Table S6) and used for identifying Accumulibacter-related sequences from pyrosequencing reads by BLASTN (v. 2.2.28+)^31^. The BLASTN Best-hits were screened to identify the candidate Accumulibacter-like 16S rRNA gene sequences according to minimum identity of 97% and alignment length of 200 bp. The candidate sequences from each sample were manually checked online at NCBI to validate the identification^27^.

### Accumulibacter diversity analysis

Following the methods published by He *et al.*^8^, Shannon diversity index (H = -∑Pi ln Pi)^32^,^33^ and Pielou evenness index (R = H/lnS)^34^,^35^ were calculated to determine the clade diversity and evenness within the Accumulibacter lineage for each sample, respectively. Pi indicated the relative abundance of each clade in the Accumulibacter lineage as quantified by the qPCR-*ppk1* assay. S represented the richness value that was determined by the number of clades as detected by the qPCR-*ppk1* assay. Profiling of the Accumulibacter lineage structure was linked to the operational conditions of full-scale WWTPs, including influent COD, TN and TP, sampling temperature, and latitude of the location, according to RDA. Contribution of each set of variables was evaluated by Monte Carlo permutation tests using 1000 permutations as implemented in CANOCO (v. 4.5)^36^.

### The *ppk1* gene clone library

To characterize the Accumulibacter population in full-scale AS samples, the *ppk1* gene as the phylogenetic marker was amplified from the AS samples of CN-GZ-DT, UK-LB-LB, UK-WL-OW, JP-A2O-TK by using the primer set of ACCppk1-254F (5’-TCACCACCGACGGCAAGAC-3’) and ACCppk1-1376R (5’-ACGATCATCAGCATCTTGGC-3’)^10^. Triplicate 50 μL PCR amplification solutions were prepared for each sample, containing 25 μL of Premix Ex Taq^TM^ (TaKaRa, Dalian, China), 1 μL of 10 μM forward and reverse primers, 50 ng of extracted DNA, and water (RT-PCR grade, Ambion Inc., USA). The thermocycling steps for PCR were set as follows: initial denaturation at 95 °C for 4 min followed by 30 cycles of denaturation at 95 °C for 30 s, annealing at 65 °C for 1 min, and extension at 72 °C for 2 min; and a final extension step at 72 °C for 12 min. Fifty clones from each clone library were sequenced in both directions using vector primers, and paired reads were quality- and vector-trimmed, and assembled by a custom script online (http://124.17.29.44/customer.html). To further confirm the accuracy of the novel clades sequences, the *ppk1* gene amplicons from sludge samples of CN-GZ-DT and JP-A2O-TK were sent to BGI for the clone library again. Twenty *ppk1* gene sequences were obtained from each sample to validate the accuracy of sequences. Putative chimeras were checked by using Bellerophon^37^ and manually using partial treeing analysis^11^. The obtained sequences were aligned with publicly available *ppk1* sequences to construct a phylogenetic tree by employing maximum likelihood method within MEGA (v. 5.02)^24^ and deposited in GenBank under accession numbers of KP737870-KP738109.

### Data availability

The draft genome sequences of *Candidatus* Accumulibacter Clade IB HKU-1 and Clade IIC HKU-2 have been deposited in the NCBI whole genome shotgun database under the accession numbers of LBIU00000000 and LBIV00000000, respectively.

## Additional Information

**How to cite this article**: Mao, Y. *et al.* Dominant and novel clades of *Candidatus* Accumulibacter phosphatis in 18 globally distributed full-scale wastewater treatment plants. *Sci. Rep.* **5**, 11857; doi: 10.1038/srep11857 (2015).

## Supplementary Material

Supplementary Information

## Figures and Tables

**Table 1:** Characteristics of 18 wastewater treatment plants.

ID	WWTP		Percentage (%) of municiple wastewater	Process	Flow rate (10^3^ m^3^/d)	Average (or range) (mg/L)			Latitude	Longitude	Temperature (^o^C)		Sampling time (mm/year)
	Code	Full name				COD	TN	TP			Range	At sampling	
1	**UK-WL-OW**	Wanlip Old Works (Leicester, UK)	Predominant	A/A/O	95	657	38	8	N52.4	W1.6	10–20	NA^b^	**11/2012**
2	**UK-NW-NW**	Crankley Point (Newark, UK)	Significant trade	CAS	11	1302	34	6.5	N53.0	E0.5	10–20	NA	**11/2012**
3	**UK-SB-SB**	Stoke Bardolph (Nottingham, UK)	Predominant	CAS	142	532	35	6.2	N52.6	W1.2	10–20	NA	**11/2012**
4	**UK-LB-LB**	Loughborough (Loughborough, UK)	Predominant	A/O + RAS fermentation	**23**	452	34	5.5	N52.5	W1.1	10–20	NA	**11/2012**
5	**US-CO-CO**	Columbia regional(Columbia, USA)	Predominant	CAS	76	700 (500–1100)	32 (21–42)	NA	N38.9	W92.2	11–23	17	04/2010
6	**US-GR-PC**	Potato Creek (Griffin, USA)	Predominant	OD	8	402	30	4.0	N33.2	W84.2	15–20	18	05/2010
7	**CA-GP-GP**	Guelph (Guelph, Canada)	Predominant	CAS	55	270	45	7.0	N43.5	W80.2	13–23	13	01/2010
8	**JP-A2O-TK**^a^	(Tsukuba, Japan)	Predominant	A/A/O	10	102	37	3.9	N36.1	E140.1	11–24	19	09/2012
9	**JP-STD-TK**^a^	(Tsukuba, Japan)	Predominant	CAS	14	102	37	3.9	N36.1	E140.1	11–24	19	09/2012
10	**SG-SG-UP**	Ulu Pandan (Singapore)	Predominant	CAS + MBR	23	265	45	9.2	N1.3	E103.6	23–32	27	05/2010
11	**CN-BJ-BX**	Bei-Xiao-He (Beijing, PRC)	95	A/A/O + MBR	100	462	51	9.3	N39.9	E116.4	16–25	16	12/2009
12	**CN-NJ-SJ**	Suo-Jin-Cun (Nanjing, PRC)	85	A/O	15	330	57	5.8	N32.1	E118.6	16–29	22	11/2009
13	**CN-SH-MH**	Min-Hang (Shanghai, PRC)	70	A/O	44	267 (140–576)	40 (34–53)	2.9 (2–6)	N31.2	E121.3	12–28	16	11/2009
14	**CN-GZ-DT**	Da-Tan-Sha (Guangzhou, PRC)	60	A/A/O	150	595	55	5.2	N23.1	E113.2	20–30	26	04/2010
15	**CN-WH-LW**	Long-Wang-Zui (Wuhan, PRC)	Predominant	A/A/O	161	277	23	4.9	N30.5	E114.3	16–29	17	12/2009
16	**CN-QD-TD**	Tuan-Dao (Qingdao, PRC)	70	A/O	100	900	120	10.0	N36.1	E120.2	12–20	17	12/2009
17	**CN-HK-ST**	Sha-Tin (Hong Kong, PRC)	95	A/O	216	226–491	26–40	3.1–5.3	N22.3	E114.2	22–32	27	11/2009
18	**CN-HK-SH**	Shek-Wu-Hui (Hong Kong, PRC)	90	A/O	82	206–529	28–41	3.0–5.6	N22.3	E114.1	21–31	30	08/2010

Abbreviations: WWTP, wastewater treatment plant; COD, chemical oxygen demand; TN, total nitrogen; TP, total phosphorus; CAS, conventional activated sludge; A/A/O, anaerobic/anoxic/aerobic; A/O, anoxic/aerobic; MBR, membrane bioreactor; OD: oxidation ditch.

^a^JP-A2O-TK and JP-STD-TK were from one sewage treatment plant in Tsukuba (Japan), but the activated sludge samples were extracted from two different treatment pipelines using A/A/O process and CAS process respectively.

^b^NA indicated not applicable. The temperatures at sampling UK-WL-OW, UK-NW-NW, UK-SB-SB, UK-LB-LB were assumed to be 15 °C for the correlation analysis according to the average temperature in the WWTPs.

**Table 2:** Relative distributions of Accumulibacter clades and estimated abundance of the total Accumulibacter lineage relative to the bacterial community.

Sample	Relative abundance (%) of indicated clade within the Accumulibacter lineage^a^					Percentage (%) of Accumulibacter lineage within bacterial community		
	I	IIA	IIB	IIC	IID	qPCR->*ppk1*^b^	qPCR-16S^c^	454-16S^d^
UK-WL-OW	**61.3 ± 2.7**	4.3 ± 2.4	9.3 ± 1.5	2.9 ± 1.4	22.3 ± 4.0	2.7 ± 0.2	3.3 ± 0.1	ND
UK-NW-NW	5.2 ± 0.5	\	10.8 ± 1.5	3.4 ± 2.3	**79.6 ± 4.7**	1.8 ± 0.2	1.9 ± 0.1	ND
UK-SB-SB	26.6 ± 6.3	7.0 ± 1.1	3.5 ± 2.3	**62.6 ± 8.3**	\	0.1 ± 0.1	0.1 ± 0.0	ND
UK-LB-LB	**50.1 ± 2.4**	15.3 ± 3.5	16.3 ± 2.3	3.1 ± 1.6	15.2 ± 3.8	1.6 ± 0.3	2.0 ± 0.1	ND
US-CO-CO	7.8 ± 2.4	2.1 ± 1.4	**69.1 ± 8.3**	2.7 ± 0.6	18.4 ± 4.3	0.3 ± 0.1	0.7 ± 0.2	0.4
US-GR-PC	2.6 ± 1.7	38.7 ± 3.5	19.2 ± 7.4	**39.6 ± 5.1**	\	0.6 ± 0.4	1.5 ± 0.3	1.6
CA-GP-GP	\	\	\	\	\	\	\	0.0
JP-A2O-TK	2.4 ± 0.7	\	40.7 ± 5.7	**52.1 ± 6.7**	4.8 ± 2.3	1.2 ± 0.3	1.5 ± 0.1	ND
JP-STD-TK	\	**60.7 ± 2.0**	11.5 ± 4.7	25.8 ± 1.5	\	0.5 ± 0.2	0.8 ± 0.3	ND
SG-SG-UP	**52.4 ± 2.5**	2.7 ± 0.3	6.8 ± 4.8	8.6 ± 4.1	29.4 ± 1.6	7.0 ± 0.3	14.2 ± 0.4	1.6
CN-BJ-BX	14.1 ± 3.9	2.0 ± 0.9	18.2 ± 3.0	26.4 ± 1.5	**39.3 ± 1.9**	0.3 ± 0.2	1.1 ± 0.1	1.3
CN-NJ-SJ	\	\	\	0.9 ± 0.5	**99.0 ± 0.6**	0.9 ± 0.0	0.9 ± 0.5	1.0
CN-SH-MH	\	36.0 ± 2.5	10.8 ± 4.5	**44.1 ± 2.7**	7.8 ± 3.5	0.9 ± 0.3	2.4 ± 0.4	0.4
CN-GZ-DT	\	26.1 ± 5.9	21.9 ± 5.0	**30.2 ± 2.8**	21.0 ± 0.1	1.3 ± 0.3	4.7 ± 0.7	1.2
CN-WH-LW	\	**52.1 ± 1.8**	34.9 ± 3.0	12.8 ± 5.0	\	3.9 ± 0.2	7.9 ± 1.9	1.2
CN-QD-TD	\	\	12.3 ± 3.0	16.1 ± 2.2	**70.6 ± 5.0**	0.4 ± 0.2	9.9 ± 2.2	1.1
CN-HK-ST	14.6 ± 1.7	\	\	23.4 ± 5.7	**62.0 ± 0.2**	0.02 ± 0.0	1.0 ± 0.0	0.1
CN-HK-SH	\	\	28.2 ± 2.9	**68.6 ± 5.4**	2.1 ± 0.0	0.5 ± 0.2	3.1 ± 0.2	0.4

^a^The relative abundance of specific clade within Accumulibacter lineage was obtained by dividing the *ppk1* copy number of each clade by the sum of the *ppk1* copy numbers from five clades. The numbers in bold indicated the dominant clade among the five clades in each sludge sample. “\” indicated that no effective amplification were detected in qPCR assays or visualized by the gel electrophoresis.

^b^Percentage of Accumulibacter lineage within bacterial community was calculated by copy numbers of *ppk1* genes from five clades and copy numbers of bacterial 16S rRNA genes.

^c^Percentage of Accumulibacter lineage within bacterial community was calculated by copy numbers of 16S rRNA genes from Accumulibacter and copy numbers of bacterial 16S rRNA genes.

^d^Percentage of Accumulibacter lineage within bacterial community was calculated according to 16S rRNA gene pyrosequencing reads assigned to Accumulibacter and those assigned to bacteria. Sludge samples from 12 WWTPs had been conducted 16S rRNA gene pyrosequencing targeting the V4 region^19^. ND, not determine.
