# Supplementary material for: Dominant and novel clades of Candidatus Accumulibacter phosphatis in 18 globally distributed full-scale wastewater treatment plants
Source: Sci Rep. 2015 Jul 3;5:11857. doi: 10.1038/srep11857 (PMC4490554; doi:10.1038/srep11857)
Supplement: Supplementary Information [file srep11857-s1.pdf]

Supplementary Information

Dominant and novel clades of *Candidatus* Accumulibacter phosphatis in 18 globally distributed full-scale wastewater treatment plants

Yanping Mao[1], David W. Graham[2], Hideyuki Tamaki[3], Tong Zhang[1]*

[1]Environmental Biotechnology Laboratory, Department of Civil Engineering, The University of Hong Kong, Pokfulam Road, Hong Kong

[2]School of Civil Engineering and Geosciences, Newcastle University, Newcastle upon Tyne NE1 7RU, United Kingdom

[3]Bioproduction Research Institute, National Institute of Advanced Industrial Science and Technology (AIST), Tsukuba, Japan

[*]Corresponding author: Tong Zhang, Environmental Biotechnology Laboratory, Department of Civil Engineering, The University of Hong Kong, Pokfulam Road, Hong Kong. E-mail: zhangt@hku.hk

Figure and Table Captions

Table S1. Optimal primers information and qPCR conditions for this study.

Table S2. Relative distribution (%) of indicated clade of Accumulibacter in the bacterial communities according to the qPCR analysis using *ppk1* gene as biomarker.

Table S3. Relative distribution (%) of indicated clade within the Accumulibacter lineage by estimating the total abundance of the Accumulibacter lineage based on the qPCR assays targeting the 16S rRNA genes.

Table S4. Summary statistics for redundancy analysis based on the relative abundances of different Accumulibacter clades in the bacterial communities.

Table S5. Summary statistics for redundancy analysis based on the relative abundances of different Accumulibacter clades in the Accumulibacter lineage.

Table S6. Reference 16S rRNA gene sequences of Accumulibacter lineage.

Figure S1. Validation curves plotting the dilution of sludge samples against the Ct values. PCR amplification efficiencies were calculated according to the equation: $E= 10^{-1/slope} -1$ to validate whether the sludge samples contained substances that inhibit PCR.

Figure S2. Phylogenetic tree of *ppk1* gene sequences from PCR amplification products using primer sets targeting a) clade IA, b) clade IIA, c) clade IIB, d) clade IIC and e) clade IID in the Accumulibacter lineage.

Figure S3. Maximum likelihood phylogenetic tree of Accumulibacter *ppk1* gene sequences. Over 360 of published *ppk1* nucleotide reference sequences[2] were downloaded from NCBI

database. Sequences belonging to Accumulibacter clades IIH and II-I are from the clone libraries. The node labels refer to bootstrap support values.

Figure S4. Agarose gel electrophoresis images of the qPCR products targeting the *ppk1* genes in clades IIB and IIC respectively. Plasmids containing specific copy numbers of *ppk1* genes were used for drawing the standard curves. Some *ppk1* genes amplified from the activated sludge samples by using the *ppk1* gene primer sets were visualized. Sterile water was used for the negative control.

Table S1. Optimal primers information and qPCR conditions for this study.

Target	Primer	Sequence (5' – 3')	T[a] (℃)	Primer concentration (nM)	Standard curve correlation efficient (R^2)	PCR efficiency (%)
Acc-IA *ppk1*	Acc-ppk1-763f	GACGAAGAAGCGGTCAAG	61	500	0.99 ± 0.01	105 ± 2
	Acc-ppk1-1170r	AACGGTCATCTTGATGGC				
Acc-IIA *ppk1*	Acc-ppk1-893f	AGTTCAATCTCACCGAGAGC	61	400[a]	0.98 ± 0.01	92 ± 5
	Acc-ppk1-997r	GGAACTTCAGGTCGTTGC				
Acc-IIB *ppk1*	Acc-ppk1-870f	GATGACCCAGTTCCTGCTCG	61	400	0.98 ± 0.01	88 ± 2
	Acc-ppk1-1002r	CGGCACGAACTTCAGATCG				
Acc-IIC *ppk1* excluding OTU NS D3[b]	Acc-ppk1-1123f	GAACAGTCCGCCAACGACC	63	500	0.97 ± 0.01	97 ± 5
	Acc-ppk1-1376r	ACGATCATCAGCATCTTGGC				
Acc-IID *ppk1*	Acc-ppk1-375f	GGGTATCCGTTTCCTCAAGCG	63	400	0.97 ± 0.01	97 ± 4
	Acc-ppk1-522r	GAGGCTCTTGTTGAGTACACGC				
Acc 16S rRNA genes	518f	CCAGCAGCCGCGGTAAT	65	400	0.99 ± 0.01	106 ± 1
	846r	GTTAGCTACGGCACTAAAAGG				
Bacterial 16S rRNA genes	341f	CCTACGGGAGGCAGCAG	60	400	0.98 ± 0.01	87 ± 1
	534r	ATTACCGCGGCTGCTGG				

[a] Modified qPCR condition from previously published paper [1].

[b] The relative abundance of Accumulibacter in Clade IIC was estimated by the qPCR assay using primer sets targeting Accumulibacter IIC excluding OTU NS D3 [1].

Table S2. Relative distribution (%) of indicated clade of Accumulibacter in the bacterial communities according to the qPCR analysis using *ppk1* gene as biomarker.

Sample	I	IIA	IIB	IIC	IID
UK-WL-OW	**1.64**	0.11	0.25	0.08	0.60
UK-NW-NW	0.10	0.00	0.20	0.06	**1.45**
UK-SB-SB	0.03	0.01	0.00	**0.06**	0.00
UK-LB-LB	**0.79**	0.24	0.26	0.05	0.24
US-CO-CO	0.02	0.01	**0.22**	0.01	0.06
US-GR-PC	0.01	0.22	0.11	**0.22**	0.00
CA-GP-GP	0.00	0.00	0.00	0.00	0.00
JP-A2O-TK	0.03	0.00	0.47	**0.60**	0.06
JP-STD-TK	0.00	**0.32**	0.06	0.14	0.00
SG-SG-UP	**3.68**	0.19	0.48	0.60	2.07
CN-BJ-BX	0.04	0.01	0.05	**0.07**	0.10
CN-NJ-SJ	0.00	0.00	0.00	0.01	**0.91**
CN-SH-MH	0.00	0.31	0.09	**0.38**	0.07
CN-GZ-DT	0.00	0.35	0.29	**0.40**	0.28
CN-WH-LW	0.00	**2.02**	1.36	0.50	0.00
CN-QD-TD	0.00	0.00	0.05	0.07	**0.31**
CN-HK-ST	0.00	0.00	0.00	0.00	**0.00**
CN-HK-SH	0.00	0.00	0.14	**0.33**	0.01

Table S3. Relative distribution (%) of indicated clade within the Accumulibacter lineage by estimating the total abundance of the Accumulibacter lineage based on the qPCR assays targeting the 16S rRNA genes.

Sample	I	IIA	IIB	IIC	IID	Other clades
UK-WL-OW	**49.2**	3.4	7.5	2.3	17.9	19.7
UK-NW-NW	5.2	0.0	10.6	3.4	**78.3**	2.5
UK-SB-SB	26.6	7.0	3.5	**62.6**	0.0	0.0
UK-LB-LB	**39.7**	12.1	12.9	2.5	12.0	20.8
US-CO-CO	3.7	1.0	32.7	1.3	8.7	**52.6**
US-GR-PC	1.0	14.6	7.2	14.9	0.0	**62.4**
CA-GP-GP	0.0	0.0	0.0	0.0	0.0	0.0
JP-A2O-TK	1.8	0.0	30.9	**39.6**	3.7	24.0
JP-STD-TK	0.0	**39.9**	7.6	17.0	0.0	35.5
SG-SG-UP	26.0	1.4	3.4	4.3	14.6	**50.5**
CN-BJ-BX	3.3	0.5	4.3	6.3	9.3	**76.3**
CN-NJ-SJ	0.0	0.0	0.0	0.9	**99.5**	0.0
CN-SH-MH	0.0	12.7	3.8	15.5	2.8	**65.3**
CN-GZ-DT	0.0	7.5	6.3	8.7	6.0	**71.6**
CN-WH-LW	0.0	25.6	17.2	6.3	0.0	**50.8**
CN-QD-TD	0.0	0.0	0.5	0.7	3.1	**95.6**
CN-HK-ST	0.1	0.0	0.0	0.2	0.5	**99.3**
CN-HK-SH	0.0	0.0	4.4	10.6	0.3	**84.7**

Table S4. Summary statistics for redundancy analysis based on the relative abundances of different Accumulibacter clades in the bacterial communities.

	Explain	P-value (focus scaling on inter-clades correlation)	P-value (focus scaling on inter-sample distance)
TP	0.17	0.08	0.08
Latitude	0.11	0.16	0.16
COD	0.04	0.42	0.42
Sampling temperature	0.04	0.63	0.63
TN	0.02	0.56	0.56

Abbreviations: TP, total phosphorus; COD, chemical oxygen demand; TN, total nitrogen.

Table S5. Summary statistics for redundancy analysis based on the relative abundances of different Accumulibacter clades in the Accumulibacter lineage.

	Explain	P-value (focus scaling on inter-clades correlation)	P-value (focus scaling on inter-sample distance)
TP	0.15	**0.04**	**0.04**
COD	0.14	**0.05**	0.06
TN	0.12	0.06	0.07
Sampling temperature	0.02	0.85	0.86
Latitude	0.01	0.98	0.98

Abbreviations: TP, total phosphorus; COD, chemical oxygen demand; TN, total nitrogen.

Table S6. Reference 16S rRNA gene sequences of Accumulibacter lineage.

PAO	Clade	Accession NO.	Ref.
Accumulibacter	I	AF204244	1
		AF255641	
		AF502224	
		AF502225	
		AJ224937	
		AY064178	
		AY064179	
		EF565148	
	IIA	AF204247	
		AF502227	
		AF502229	
		AF502230	
		AF502231	
		EF565147	
		EF565153	
		EF565155	
		EF565156	
	IIB	EF565149	
		EF565150	
		EF565152	
		EF565157	
	IIC & IID	AY062125	
		EF565151	
		EF565158	
		EF565159	
		EF565160	
		EF565161	

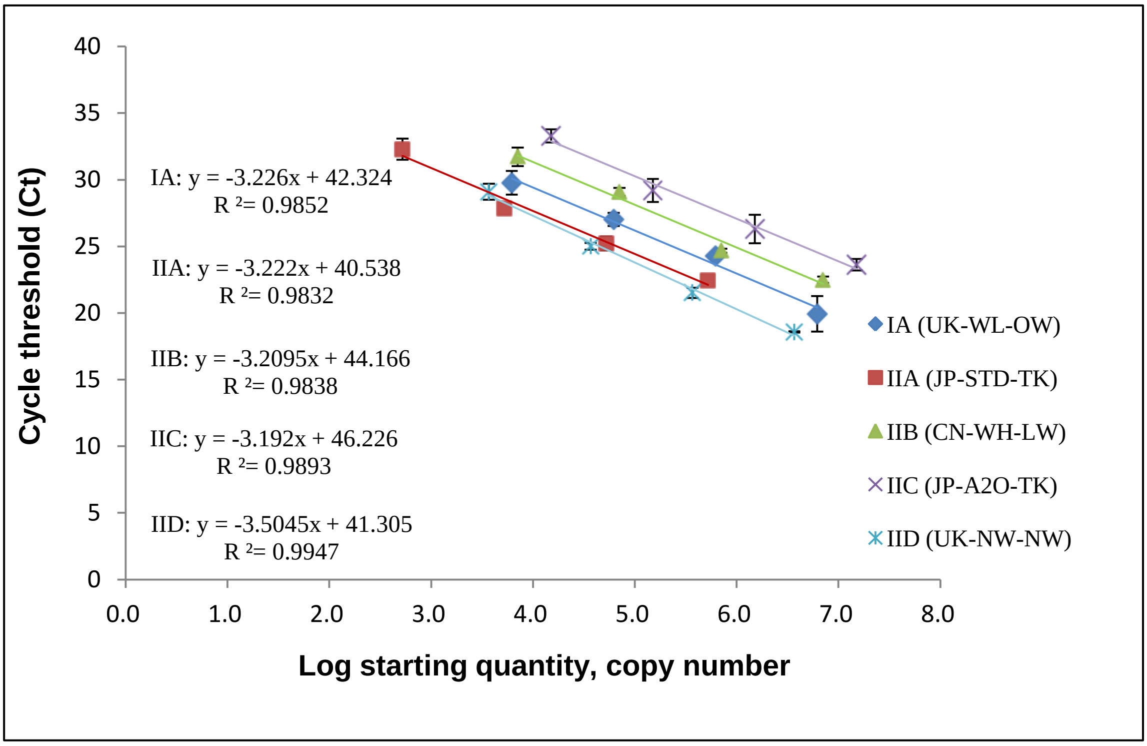

Figure S1. Validation curves plotting the dilution of sludge samples against the Ct values. PCR amplification efficiencies were calculated according to the equation: $E= 10^{-1/slope} -1$ to validate whether the sludge samples contained substances that inhibit PCR.

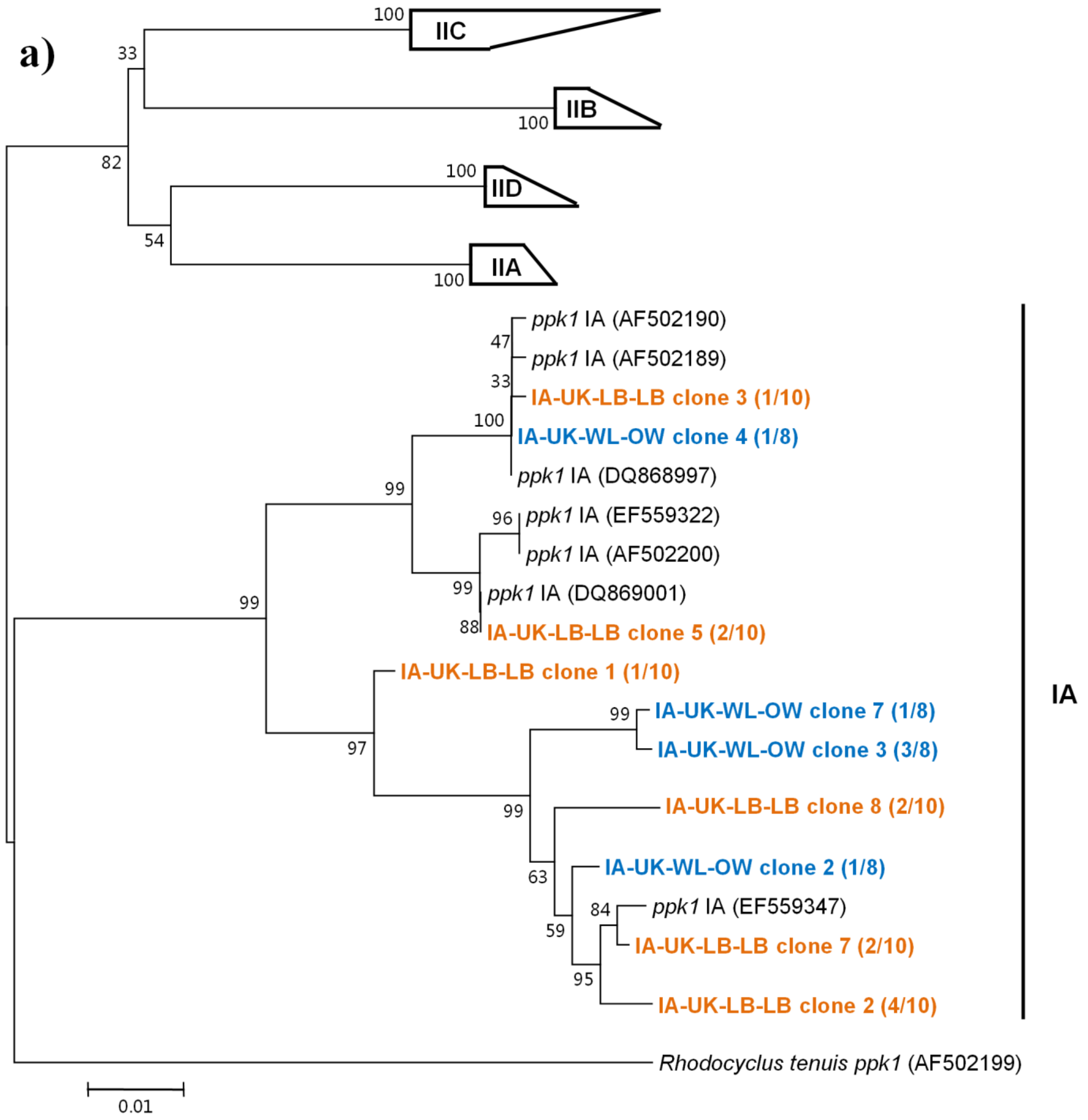
a)
IIC
IIB
IID
IIA
ppk1 IA (AF502190)
ppk1 IA (AF502189)
IA-UK-LB-LB clone 3 (1/10)
IA-UK-WL-OW clone 4 (1/8)
ppk1 IA (DQ868997)
ppk1 IA (EF559322)
ppk1 IA (AF502200)
ppk1 IA (DQ869001)
IA-UK-LB-LB clone 5 (2/10)
IA-UK-LB-LB clone 1 (1/10)
IA-UK-WL-OW clone 7 (1/8)
IA-UK-WL-OW clone 3 (3/8)
IA-UK-LB-LB clone 8 (2/10)
IA-UK-WL-OW clone 2 (1/8)
ppk1 IA (EF559347)
IA-UK-LB-LB clone 7 (2/10)
IA-UK-LB-LB clone 2 (4/10)
Rhodocyclus tenuis ppk1 (AF502199)
IA
0.01

b)

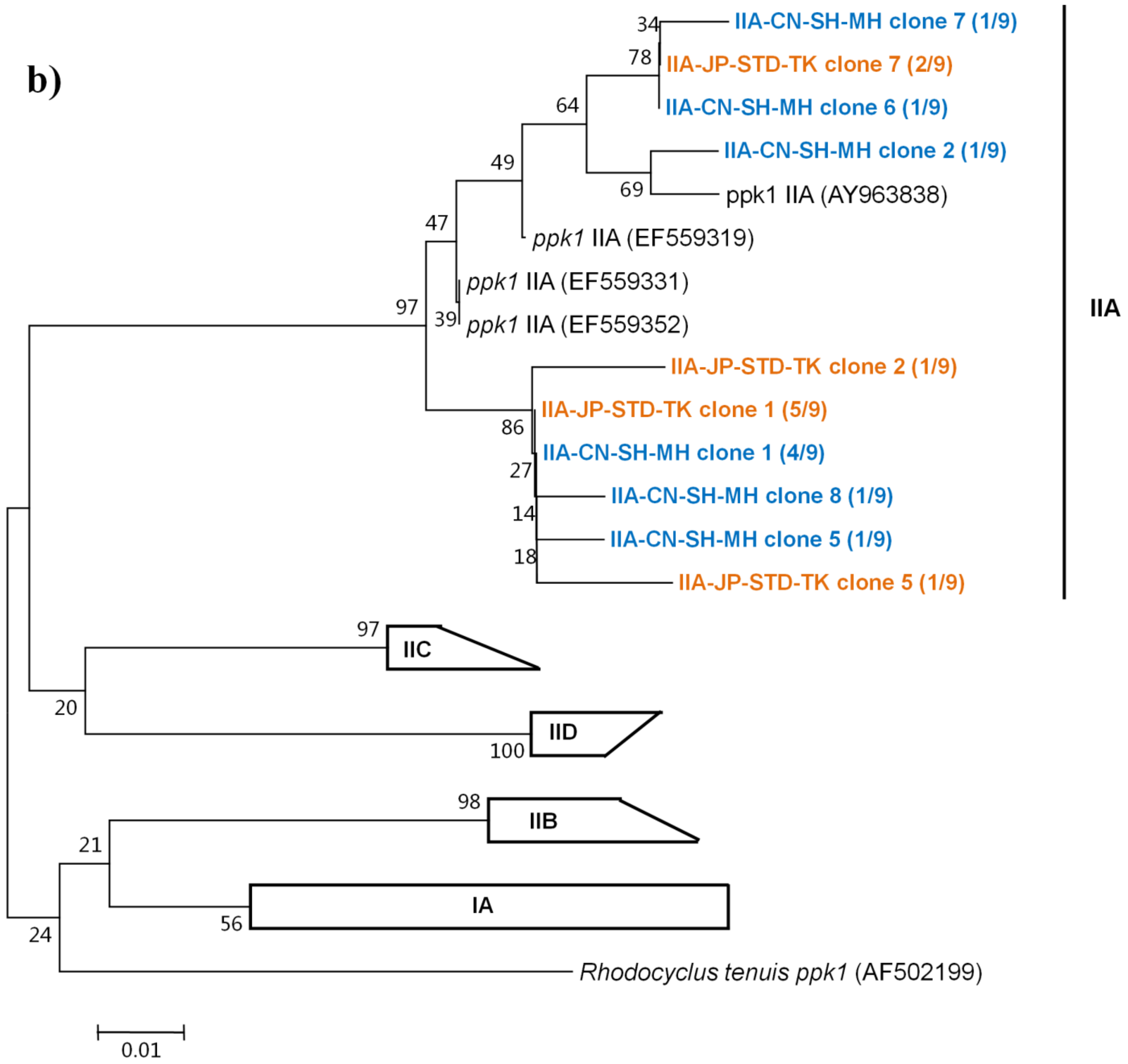
IIA-CN-SH-MH clone 7 (1/9)
IIA-JP-STD-TK clone 7 (2/9)
IIA-CN-SH-MH clone 6 (1/9)
IIA-CN-SH-MH clone 2 (1/9)
ppk1 IIA (AY963838)
ppk1 IIA (EF559319)
ppk1 IIA (EF559331)
ppk1 IIA (EF559352)
IIA-JP-STD-TK clone 2 (1/9)
IIA-JP-STD-TK clone 1 (5/9)
IIA-CN-SH-MH clone 1 (4/9)
IIA-CN-SH-MH clone 8 (1/9)
IIA-CN-SH-MH clone 5 (1/9)
IIA-JP-STD-TK clone 5 (1/9)
IIA
IIC
IID
IIB
IA
Rhodocyclus tenuis ppk1 (AF502199)
34
78
64
49
69
47
97
39
86
27
14
18
97
20
100
98
21
56
24
0.01

c)

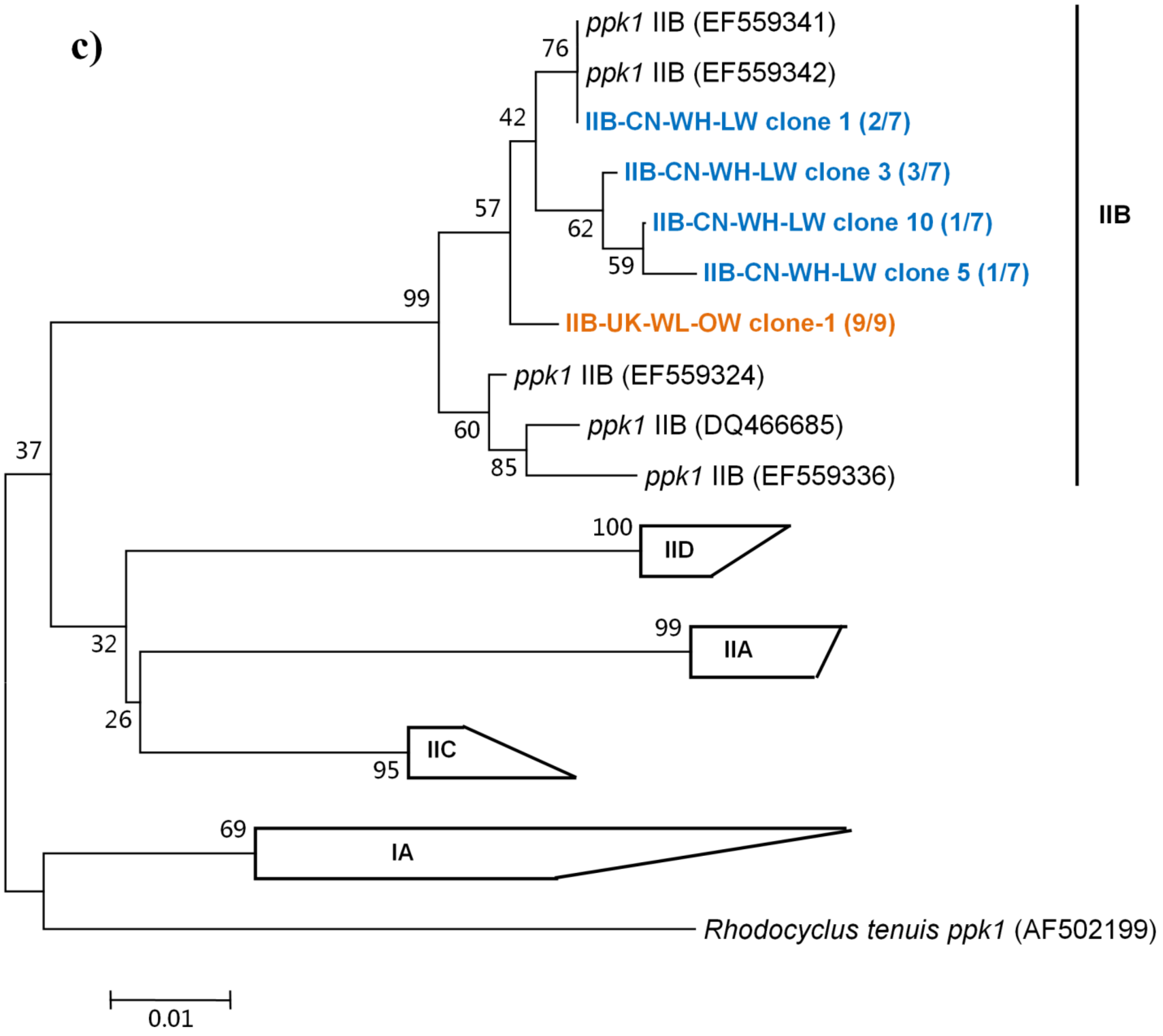

ppk1 IIB (EF559341)
ppk1 IIB (EF559342)
IIB-CN-WH-LW clone 1 (2/7)
IIB-CN-WH-LW clone 3 (3/7)
IIB-CN-WH-LW clone 10 (1/7)
IIB-CN-WH-LW clone 5 (1/7)
IIB-UK-WL-OW clone-1 (9/9)
ppk1 IIB (EF559324)
ppk1 IIB (DQ466685)
ppk1 IIB (EF559336)
IIB
76
42
57
62
59
99
60
85
37
100
IID
99
IIA
32
26
IIC
95
69
IA
Rhodocyclus tenuis ppk1 (AF502199)
0.01

d)

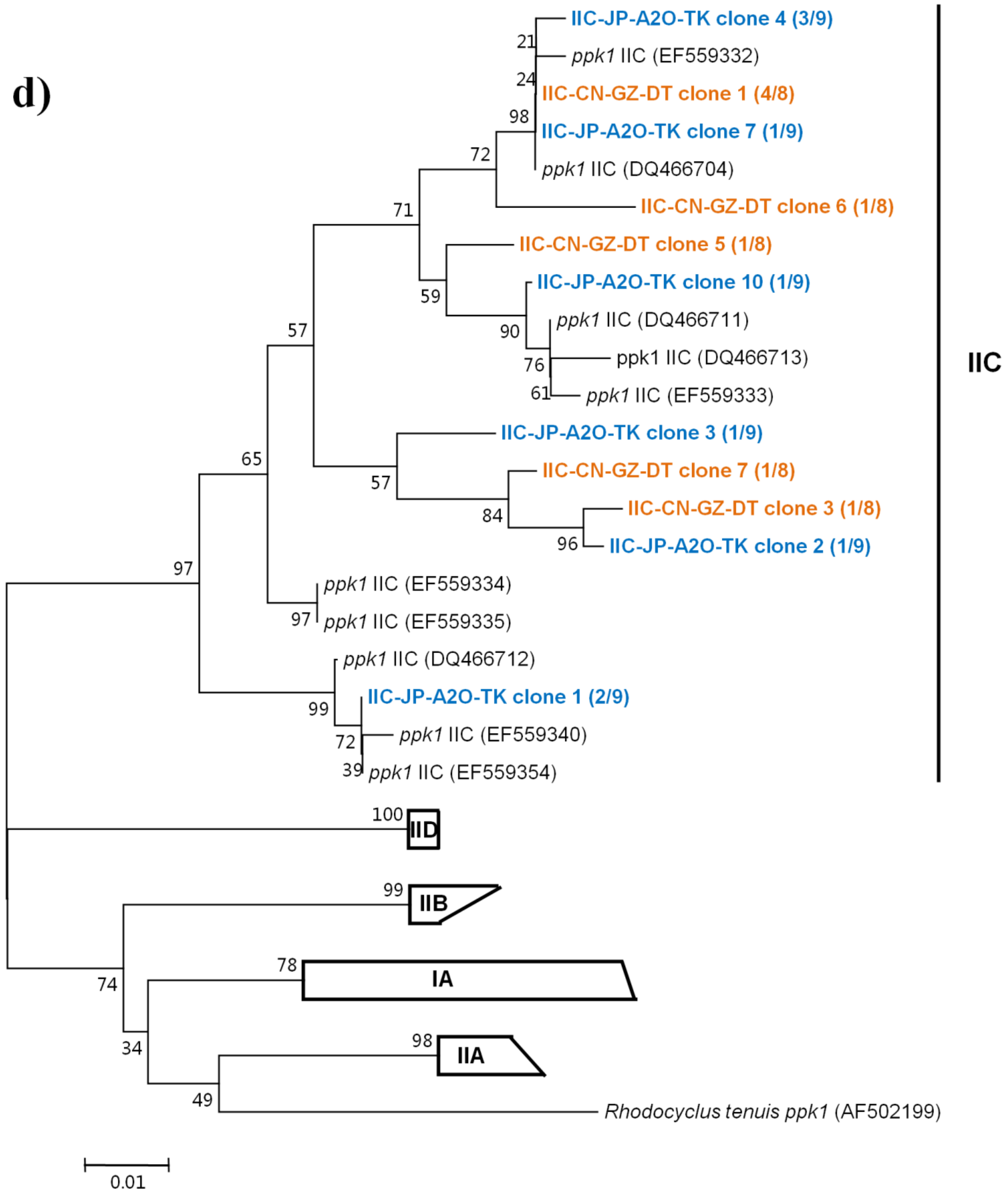

IIC-JP-A2O-TK clone 4 (3/9)
ppk1 IIC (EF559332)
IIC-CN-GZ-DT clone 1 (4/8)
IIC-JP-A2O-TK clone 7 (1/9)
ppk1 IIC (DQ466704)
IIC-CN-GZ-DT clone 6 (1/8)
IIC-CN-GZ-DT clone 5 (1/8)
IIC-JP-A2O-TK clone 10 (1/9)
ppk1 IIC (DQ466711)
ppk1 IIC (DQ466713)
ppk1 IIC (EF559333)
IIC-JP-A2O-TK clone 3 (1/9)
IIC-CN-GZ-DT clone 7 (1/8)
IIC-CN-GZ-DT clone 3 (1/8)
IIC-JP-A2O-TK clone 2 (1/9)
ppk1 IIC (EF559334)
ppk1 IIC (EF559335)
ppk1 IIC (DQ466712)
IIC-JP-A2O-TK clone 1 (2/9)
ppk1 IIC (EF559340)
ppk1 IIC (EF559354)
IIC
IID
IIB
IA
IIA
Rhodocyclus tenuis ppk1 (AF502199)
0.01

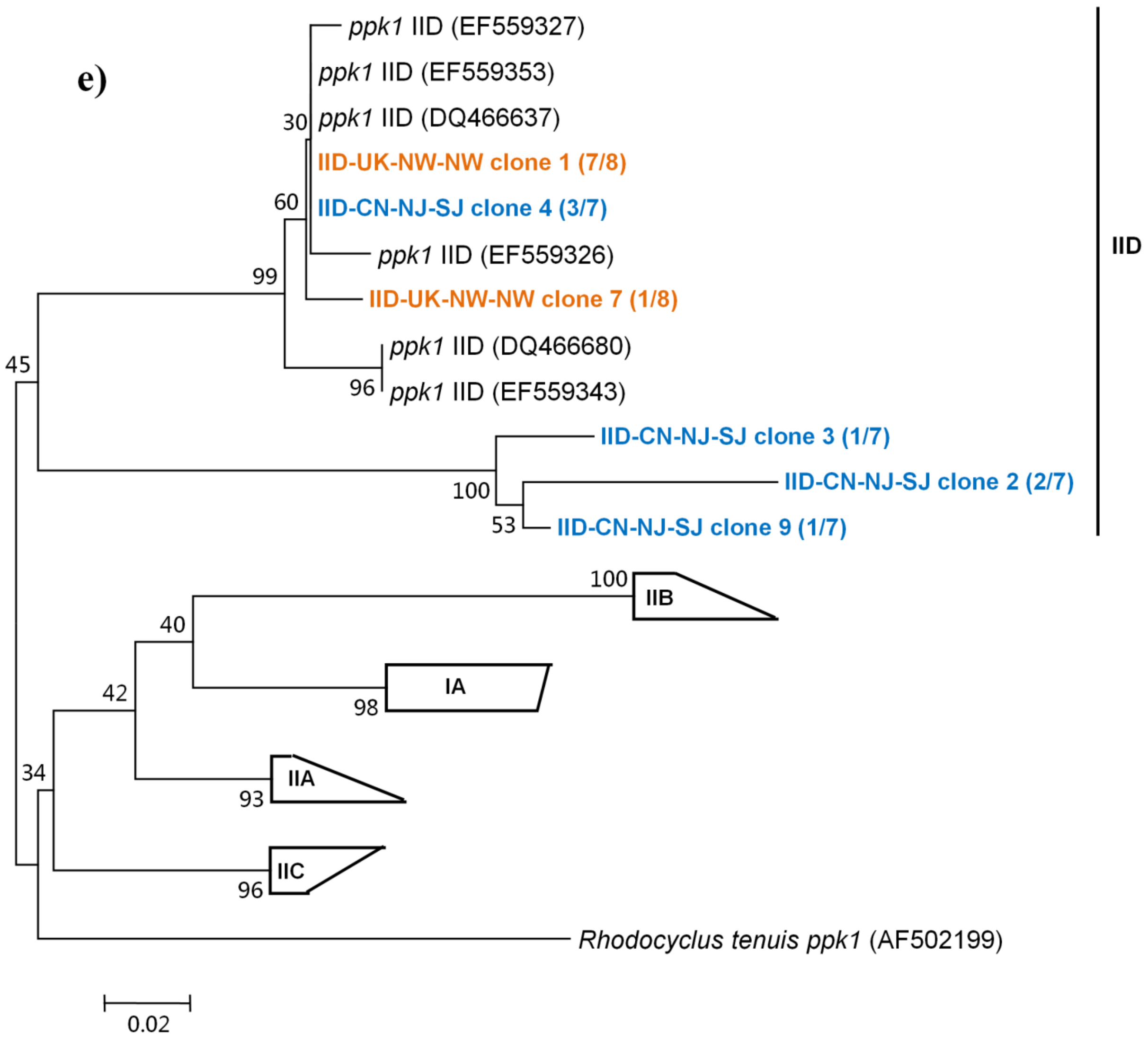

Figure S2. Phylogenetic tree of *ppk1* gene sequences from PCR amplification products using primer sets targeting a) clade IA, b) clade IIA, c) clade IIB, d) clade IIC and e) clade IID in the Accumulibacter lineage.

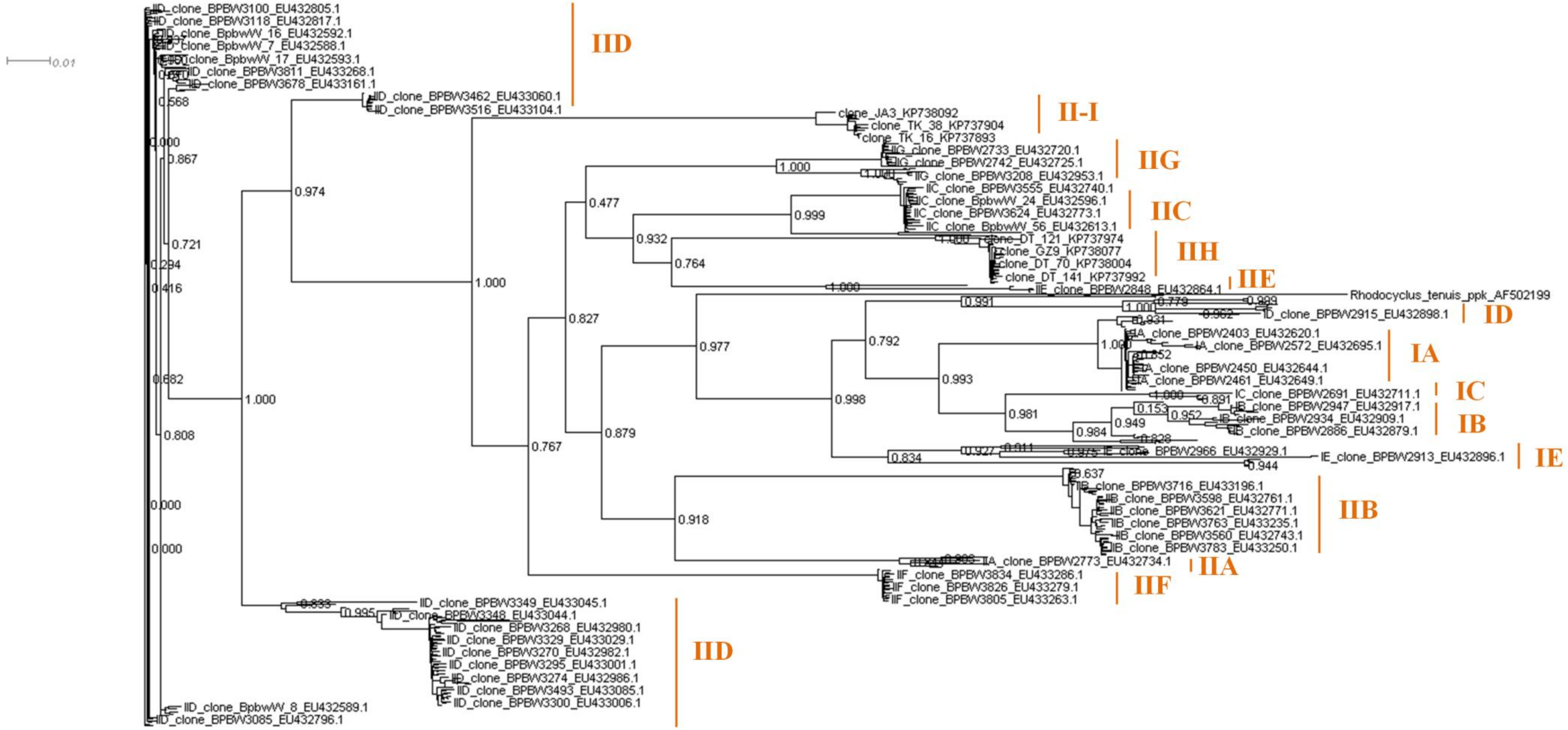

Figure S3. Maximum likelihood phylogenetic tree of Accumulibacter *ppk1* gene sequences. Over 360 of published *ppk1* nucleotide reference sequences[2] were downloaded from NCBI database. Sequences belonging to Accumulibacter clades IIH and II-I are from the clone libraries. The node labels refer to bootstrap support values.

qPCR results targeting *ppk1* genes in clade IIB

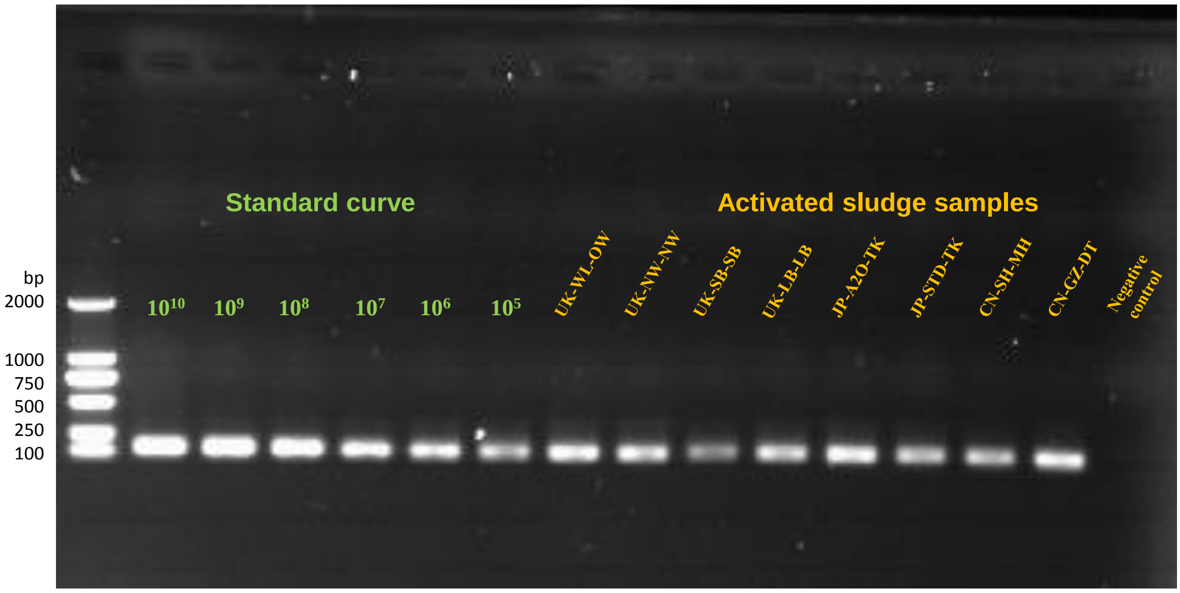

qPCR results targeting *ppk1* genes in clade IIC

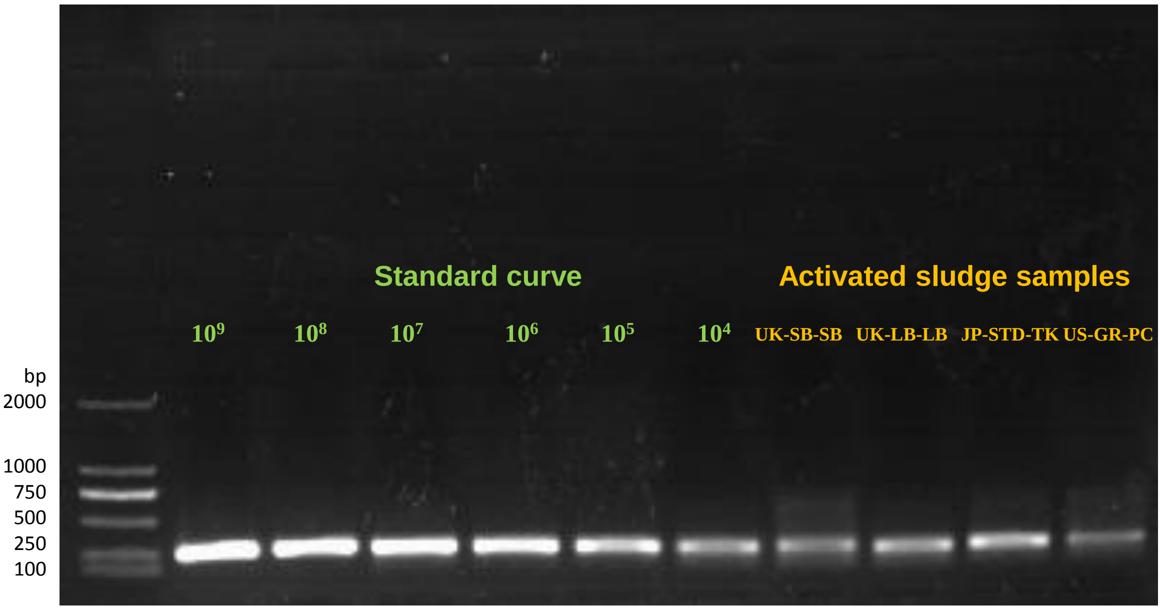

Figure S4. Agarose gel electrophoresis images of the qPCR products targeting the *ppk1* genes in clades IIB and IIC respectively. Plasmids containing specific copy numbers of *ppk1* genes were used for drawing the standard curves. Some *ppk1* genes amplified from the activated sludge samples by using the *ppk1* gene primer sets were visualized. Sterile water was used for the negative control.

REFERENCE

1. He, S., Gall, D. L. & McMahon, K. D. "*Candidatus* Accumulibacter" population structure in enhanced biological phosphorus removal sludges as revealed by polyphosphate kinase genes. *Appl. Environ. Microbiol.* **73**, 5865-5874, doi:10.1128/aem.01207-07 (2007).

2. Peterson, S. B., Warnecke, F., Madejska, J., McMahon, K. D. & Hugenholtz, P. Environmental distribution and population biology of *Candidatus* Accumulibacter, a primary agent of biological phosphorus removal. *Environ. Microbiol.* **10**, 2692-2703, doi:10.1111/j.1462-2920.2008.01690.x (2008).
